# Renal Dose Adjustment in European Primary Care: Clinical Nuances and Practical Challenges

**DOI:** 10.3390/jcm15124737

**Published:** 2026-06-18

**Authors:** Anna Maria Dworakowska, Jolanta Małyszko, Magdalena Bujalska-Zadrożny

**Affiliations:** 1 Department of Pharmacotherapy and Pharmaceutical Care, Faculty of Pharmacy, Medical University of Warsaw, 02-097 Warsaw, Poland; 2 Department of Nephrology, Dialysis and Internal Medicine, Faculty of Medicine, Medical University of Warsaw, 02-097 Warsaw, Poland; jolanta.malyszko@wum.edu.pl

**Keywords:** CKD, dose adjustment, medicines optimization, primary care, renal dosing

## Abstract

Appropriate dose adjustment of renally eliminated medicines is central to safe pharmacotherapy in patients with chronic kidney disease; yet, in European primary care, it is systematically undermined not by lack of knowledge, but by structural misalignment between laboratory reporting, regulatory product information, and clinical guidelines. This Perspective argues that the core barrier to optimal renal dose adjustment is a mismatch between routinely reported indexed eGFR and dosing requirements based on absolute renal function, compounded by persistent regulatory reliance on the Cockcroft–Gault equation despite its known limitations. We show how these structural inconsistencies, together with patient-related factors such as frailty, ageing, and body size, generate uncertainty at the point of prescribing and contribute to persistent variability in dosing decisions. To address this challenge, we propose a structured, context-aware renal dosing framework designed for routine primary care. The framework integrates regulatory guidance, multiple methods of renal function estimation, and patient-specific modifiers into a stepwise decision process. Clinical vignettes illustrate how divergent renal function estimates and regulatory requirements can lead to different dosing decisions in everyday practice. By reframing renal dose adjustment as a context-driven clinical process rather than a purely equation-based task, this Perspective highlights the need for regulatory alignment and pragmatic decision tools to improve prescribing quality in patients with chronic kidney disease.

## 1. Introduction

Chronic kidney disease (CKD) represents a major and growing public health challenge in Europe, affecting an estimated 100 million individuals, largely driven by population ageing and the increasing prevalence of diabetes, hypertension, and obesity [1]. Impaired renal function profoundly influences drug disposition—via reduced excretion, altered absorption and metabolism, and uremic toxin accumulation [2]—heightening risks of toxicity or undertreatment [3,4], especially in patients with multimorbidity and polypharmacy, where renal impairment intersects with competing therapeutic priorities.

Despite the availability of dosing guidelines and reference sources [4], inappropriate prescribing in patients with CKD remains common [5]. This suggests that the problem is not solely a lack of knowledge [3,4], but reflects deeper structural and methodological inconsistencies in how renal function is assessed and translated into dosing decisions.

We argue that the central challenge of renal dose adjustment in European primary care is a systemic misalignment between three domains: laboratory reporting practices [6,7], regulatory medicinal product information [8,9], and clinical dosing requirements [3,10]. In routine practice, laboratories predominantly report estimated glomerular filtration rate (eGFR) indexed to body surface area, whereas drug dosing decisions require absolute renal function expressed in mL/min [3,10]. At the same time, the Summary of Product Characteristics (SmPC)—the legally binding reference for medicinal product use in Europe [8,9]—often relies on legacy pharmacokinetic data based on Cockcroft–Gault-derived creatinine clearance, with variable clarity, completeness, and consistency of dosing recommendations [3,11].

These discrepancies are embedded within highly heterogeneous health system structures, with variation in governance, organization, workforce capacity, and scope of practice [12,13]. In the context of renal dose adjustment, these systemic differences manifest as variability in the content and regulatory use of SmPC, which remain insufficiently harmonized across authorization pathways in Europe [14], as well as inconsistencies in laboratory reporting of renal function, including differences in equations used, indexing practices, and implementation of current recommendations [6,7]. Access to cystatin C testing also remains uneven across primary care settings, limiting the practical application of guideline-endorsed approaches to renal function assessment [6]. In parallel, the role of clinical pharmacists in medication review and dose optimization is expanding across Europe but remains heterogeneously implemented, with substantial variation in scope of practice, integration into primary care teams, and availability of structured pharmaceutical services [15]. As a result, clinicians are frequently required to reconcile discordant information sources, including inconsistently structured SmPC recommendations, differing renal function estimates, and evolving guideline standards [8,9,16,17].

Importantly, this misalignment is not incidental but reflects structural features of the European regulatory framework. Variability in SmPC content across authorization pathways has been well documented [14], and many dosing recommendations have not been systematically updated to reflect contemporary approaches to renal function estimation [18]. Although regulatory and guideline bodies—including the European Medicines Agency (EMA) and KDIGO—advocate for explicit specification of renal function estimation methods and the use of non-indexed values for dose adjustment [3,10], these principles are not consistently operationalized in clinical practice or regulatory documents. The content of SmPCs—particularly for products authorized outside the centralized procedure—may be incomplete, outdated, or insufficiently clear for clinical use [19]. Kidney function is frequently described using imprecise or outdated terminology, such as “mild”, “moderate”, or “severe” renal impairment [20], and dosing recommendations may be inconsistent, clinically difficult to interpret, or entirely lacking for specific patient populations [18].

Addressing this challenge will require targeted, system-level interventions rather than general calls for harmonization. These include clearer specification of renal function metrics in SmPCs (e.g., indexed vs. absolute eGFR) [10], systematic updating of dosing recommendations for commonly used medicines, and better alignment between regulatory guidance, laboratory reporting, and contemporary nephrology standards [3,10]. Without such coordinated efforts, variability in renal dose adjustment is likely to persist despite increasing availability of clinical guidance.

This Perspective focuses on patients with stable CKD managed in European primary care, and examines how regulatory frameworks, methodological ambiguity, and real-world constraints shape dosing decisions. Clinical scenarios involving rapidly changing renal function, such as acute kidney injury or augmented renal clearance, are beyond the scope of this discussion due to the limited reliability of standard estimation equations in these settings [3,21].

We specifically examine two interrelated tensions: (1) the mismatch between routinely reported indexed eGFR and the need for absolute renal function in drug dosing, and (2) the continued regulatory reliance on the Cockcroft–Gault equation despite its known limitations. We propose that resolving these tensions is essential to improving the safety and consistency of pharmacotherapy in patients with CKD in European primary care.

## 2. Glomerular Filtration Rate

Renal elimination capacity correlates most closely with absolute GFR. Consequently, dose adjustment of medicines predominantly eliminated via the kidneys should be guided by absolute GFR rather than the categorical staging of chronic kidney disease [3,10]. Although direct measurement of GFR using exogenous filtration markers remains the reference standard [22], such methods are time-consuming, resource-intensive, and impractical for routine clinical use [23]. In everyday practice, estimated GFR (eGFR) derived from validated serum creatinine-based equations, such as CKD-EPI 2021 [24], is therefore commonly used to inform dosing decisions in patients with stable renal function [3].

However, creatinine-based eGFR may substantially overestimate or underestimate true renal function in specific clinical contexts [25]. Serum creatinine is influenced by non-GFR determinants, including muscle mass, dietary intake, and medications that inhibit tubular creatinine secretion, potentially leading to pseudo-reductions in GFR without true deterioration of kidney function [26]. In selected patient groups—such as individuals with extreme body size or liver cirrhosis—cystatin C-based estimates may offer advantages, although their interpretation is also limited by non-renal influences and restricted availability in primary care [3,27].

Clinicians should also be aware that laboratories may report eGFR values calculated using different equations, some of which are no longer recommended [28,29]. For example, the MDRD equation remains widely reported despite its known limitations and lack of endorsement in recent guidelines [30].

Recent European research indicates that discrepancies between creatinine-based GFR estimating equations often reflect how non-GFR determinants, particularly muscle mass, are modelled across the lifespan rather than true differences in filtration capacity [31,32,33]. The European Kidney Function Consortium (EKFC) equation builds on the Full Age Spectrum concept, refining age dependency to improve performance in both younger adults and older individuals, especially in European populations. Importantly, age terms in creatinine-based equations largely act as surrogates for changes in creatinine generation rather than direct modelling of age-related GFR decline, explaining why no single equation can be universally optimal across all patient groups [31].

These conceptual limitations have important clinical consequences in older populations, where sarcopenia and frailty are common. A considerable proportion of elderly individuals—depending on the population studied and the equation used—may be classified as having CKD based on fixed eGFR thresholds despite reflecting physiological renal ageing rather than pathological kidney disease [34,35,36]. In older adults, apparent CKD based on eGFR thresholds may reflect physiological renal ageing rather than progressive kidney disease. For prescribing, the key issue is not labelling alone, but whether the observed renal function estimate is sufficient to support safe dose selection, monitoring, or avoidance of potentially nephrotoxic or renally cleared drugs.

Reclassification analyses further show that staging shifts occur most frequently around commonly used decision thresholds—particularly 60 mL/min/1.73 m^2^—depending on the equation applied, reinforcing that numerical eGFR cut-offs should be interpreted cautiously when guiding medication dosing and other management decisions in primary care [36]. While eGFR values used for CKD staging are typically indexed to a body surface area of 1.73 m^2^, both KDIGO and EMA guidance emphasize that dosing decisions should rely on absolute renal function estimates expressed in mL/min [3,10]. Failure to account for this distinction may contribute to inappropriate dose selection, including both under- and overdosing, particularly in patients at the extremes of body size.

## 3. De-Indexing eGFR and Selection of a Body Surface Area Formula

Estimated GFR values reported by laboratories are typically indexed to a standard body surface area (BSA) of 1.73 m^2^, reflecting conventions established for epidemiological classification rather than drug dosing [3]. In the context of medication dose adjustment—particularly when fixed eGFR thresholds guide clinical decisions—conversion to an absolute GFR value (mL/min) may be clinically relevant [3,37]. This de-indexing process adjusts the reported eGFR according to the patient’s actual BSA and is most consequential in individuals whose body size deviates substantially from the reference value.

Use of absolute eGFR is particularly important when dosing medicines with a narrow therapeutic index or agents for which clinical efficacy depends on achieving a defined minimum plasma concentration, such as certain antimicrobial therapies [3]. In patients with a body size close to the reference range, the numerical difference between indexed and non-indexed eGFR is often small and clinically negligible; however, at the extremes of body size, failure to de-index eGFR may contribute to inappropriate dose reduction or escalation.

Several formulas are available to estimate BSA in adult patients. The Mosteller formula is commonly recommended due to its simplicity, acceptable accuracy, and widespread use in regulatory guidance and clinical decision support systems [38]. Alternative formulas, such as the DuBois and DuBois equation [39], yield comparable results in most adult populations, and differences between methods are generally unlikely to be clinically meaningful for dosing decisions [40,41]. Nevertheless, clinicians should recognize that de-indexing eGFR represents an approximation rather than a precise measure of renal drug clearance and should be interpreted alongside other patient-specific factors and clinical judgement.

Table 1 illustrates three patients with similar indexed eGFR values (56–65 mL/min/1.73 m^2^) but markedly different body surface areas.

In Patient A, a frail older woman with low BSA (1.37 m^2^), absolute eGFR is 46 mL/min, which places her below the commonly used 60 mL/min threshold in many SmPCs. Without de-indexing, her indexed eGFR of 58 mL/min/1.73 m^2^ might be misinterpreted as close to 60 and could lead to inappropriate dose escalation or failure to reduce a dose that should be lowered.

In Patient B, a tall man of normal build with high BSA (2.12 m^2^), absolute eGFR is 80 mL/min, clearly above the 60 mL/min threshold, even though his indexed eGFR is only 65 mL/min/1.73 m^2^. Failure to de-index here could lead to unnecessary dose reduction.

In Patient C, a woman with class II obesity and high BSA (2.01 m^2^), absolute eGFR is 65 mL/min, whereas her indexed eGFR is 56 mL/min/1.73 m^2^. Without de-indexing, she would be classified below the 60 mL/min threshold and might receive an unnecessarily low dose.

These examples demonstrate that de-indexing eGFR is clinically relevant not only in frail or underweight patients but also in tall individuals of normal build and in obese patients. Ignoring the distinction between indexed and absolute eGFR can result in both underdosing and overdosing, depending on the patient’s body size.

## 4. Use of the Cockcroft–Gault Equation in Medication Dosing

The Cockcroft–Gault equation continues to play a paradoxical role in renal dose adjustment: it is no longer recommended for routine estimation of kidney function [3,4], yet remains embedded in regulatory documents and drug labelling [42]. The equation estimates creatinine clearance (CrCl), which systematically overestimates true GFR due to the tubular secretion of creatinine, and therefore does not provide an accurate measure of glomerular filtration [43].

Several methodological limitations further restrict the applicability of the Cockcroft–Gault equation in contemporary practice. The formula was derived from studies conducted predominantly in men younger than 70 years with stable renal function and near-ideal body weight, using non-standardized Jaffe-based creatinine assays [44]. In contrast, modern European laboratories routinely employ IDMS-traceable enzymatic assays, and application of the Cockcroft–Gault equation without recalibration may lead to systematic overestimation of renal function, particularly in elderly individuals and in patients with reduced muscle mass, although the magnitude of this effect varies across populations and clinical settings [11]. By comparison, MDRD and CKD-EPI equations were developed and validated using standardized creatinine measurements, improving inter-laboratory comparability and the reliability of eGFR reporting [45].

The accuracy of the Cockcroft–Gault equation is particularly compromised in elderly patients and in individuals with obesity, cachexia, or unstable renal function [46]. Despite these limitations, Cockcroft–Gault-derived CrCl remains embedded in the prescribing information of several medicines and in pivotal clinical trials, necessitating its continued use in specific regulatory and therapeutic contexts [3].

In this context, the choice of body weight used in the Cockcroft–Gault equation can substantially influence estimated renal function and subsequent dosing decisions. Use of actual body weight in obese patients may overestimate renal clearance and increase toxicity risk, whereas reliance on actual body weight in frail or underweight individuals may underestimate renal function and contribute to undertreatment. While various pragmatic approaches to weight selection have been proposed [47], no universally validated strategy exists, and interpretation of Cockcroft–Gault-derived CrCl should be supported by assessment of renal function ranges [48], renal trends, body composition, and the potential clinical consequences of over- or underdosing [3,37]. A key exception is DOACs dosing, where the Cockcroft–Gault formula must always use actual body weight per product labelling and pivotal trials [49], although clinicians should interpret these recommendations in the context of jurisdiction-specific regulatory requirements and the clinical setting.

## 5. A Context-Aware Framework for Renal Dose Adjustment

Renal dose adjustment in primary care extends beyond selecting an equation and applying fixed thresholds. In patients with multimorbidity and polypharmacy, dosing decisions require integration of clinical context, regulatory constraints, and imperfect measures of renal function [3]. The persistence of potentially inappropriate prescribing in patients with reduced eGFR—particularly among older adults—highlights the limitations of purely equation-driven approaches [50,51,52]. Effective dose optimization therefore requires structured, context-aware decision-making, often supported by interprofessional collaboration, including clinical pharmacists [53,54].

In European primary care, prescribing is anchored in the SmPC, which should serve as the initial reference point, followed by structured medication review [55,56] and individualized assessment of renal function [3]. To operationalize this process, we propose a stepwise framework designed to address the misalignment between laboratory reporting, regulatory guidance, and clinical decision-making identified in this Perspective:

Step 1: Define the clinical context. Establish patient priorities [57], cardiovascular risk, comorbidities, frailty status, and risk factors affecting drug handling (e.g., dehydration, recent acute illness) [3].

Step 2: Estimate renal function. Use a validated equation (CKD-EPI 2021 preferred [3]).

Step 3: Perform medication reconciliation. Evaluate the appropriateness of each medication [56]. Identify drugs requiring dose modification, avoidance, or enhanced monitoring.

Step 4: Identify the regulatory dosing framework. Determine whether SmPC recommendations are based on eGFR (indexed or absolute), Cockcroft–Gault-derived creatinine clearance, categorical descriptors, or other parameters.

Step 5: Apply context-aware dose adjustment. Integrate SmPC guidance with the selected renal function estimate (including method and units), patient-specific modifiers, and therapeutic goals. When dosing decisions depend on thresholds or when body size deviates from standard assumptions, convert indexed eGFR to absolute values.

If Cockcroft–Gault is required, calculate creatinine clearance using an appropriate body weight, recognizing that no single approach is universally validated. An important exception is dosing of DOACs, where regulatory labelling requires the use of actual body weight [49]. When estimates lie near decision thresholds, interpret renal function as a range rather than a single value [48] and consider trends over time, body composition, and the clinical consequences of over- or underdosing [3,37]. Avoid rigid threshold-based decisions when inconsistent with the overall clinical context. Where SmPC guidance is incomplete or unclear, consult additional validated sources.

Step 6: Monitor and reassess. Adjust therapy in response to changes in renal function, clinical status, or treatment goals, and involve multidisciplinary expertise in complex cases.

This framework reframes renal dose adjustment as a process of reconciling discordant inputs rather than applying a single equation or threshold. By explicitly accounting for differences between indexed and absolute eGFR and the regulatory role of Cockcroft–Gault, it provides a pragmatic approach to navigating the structural inconsistencies that characterize renal dosing in European primary care.

To illustrate its application, the following clinical vignettes demonstrate how different estimation methods and regulatory requirements can lead to divergent dosing decisions in routine practice.

### Case Vignettes

A 63-year-old woman (height 157 cm, body weight 93 kg, BMI 37.7 kg/m^2^) with type 2 diabetes, hypertension, and atrial fibrillation is treated with empagliflozin 10 mg once daily, olmesartan 40 mg once daily, apixaban 2.5 mg twice daily, and pregabalin 150 mg three times daily. Renal function estimates vary depending on the method used: eGFR (CKD-EPI 2021) 56 mL/min/1.73 m^2^, non-indexed eGFR 63 mL/min, and Cockcroft–Gault creatinine clearance ranging from 55 mL/min (adjusted body weight) to 77 mL/min (actual body weight).

These discrepancies place the patient at or across multiple dosing thresholds, illustrating how different estimation methods and regulatory requirements can lead to divergent clinical decisions.

Example 1: Indexed eGFR as a regulatory threshold

Clinical question: Can empagliflozin be increased from 10 mg to 25 mg?

According to the SmPC, dose escalation requires eGFR ≥60 mL/min/1.73 m^2^. The recommendation is explicitly based on indexed eGFR [58].

Using the reported value (56 mL/min/1.73 m^2^), the patient does not meet the criterion, despite having a non-indexed eGFR of 63 mL/min.

Clinical decision: Do not increase the dose; continue 10 mg once daily.

This case illustrates how reliance on indexed eGFR in regulatory documents may override clinically relevant differences in absolute renal function.

Example 2: Cockcroft–Gault and body weight ambiguity

Clinical question: Is olmesartan 40 mg once daily appropriate?

The SmPC limits dosing to 20 mg daily in patients with creatinine clearance 20–60 mL/min, requiring use of the Cockcroft–Gault equation [59].

Estimated creatinine clearance varies substantially depending on body weight: 77 mL/min (actual body weight) versus 55 mL/min (adjusted body weight). For obesity, the use of actual body weight may overestimate renal function.

Clinical decision: A dose reduction to 20 mg once daily should be considered.

This example highlights how the absence of standardized guidance on weight selection within the Cockcroft–Gault equation introduces clinically relevant variability in dosing decisions.

Example 3: Regulatory lock-in of Cockcroft–Gault (DOACs)

Clinical question: Is apixaban 2.5 mg twice daily appropriate?

According to the SmPC [60], in patients with non-valvular atrial fibrillation, dose reduction to 2.5 mg twice daily is indicated in severe renal impairment (creatinine clearance 15–29 mL/min). In patients with mild-to-moderate renal impairment, dose reduction is required only if at least two of the following criteria are present: age ≥ 80 years, body weight ≤ 60 kg, or serum creatinine ≥ 1.5 mg/dL.

Renal function assessment should be based on Cockcroft–Gault creatinine clearance using actual body weight, consistent with regulatory labelling and pivotal trials [49]. In this patient, CrCl is 77 mL/min.

None of the dose-reduction criteria are met (age 63 years, body weight 93 kg, serum creatinine 1.1 mg/dL).

Clinical decision: The reduced dose (2.5 mg twice daily) is not appropriate; standard dosing (5 mg twice daily) should be used.

This case illustrates how dosing is tightly anchored to specific regulatory criteria and the mandated use of Cockcroft–Gault, limiting flexibility even when alternative renal function estimates are available.

Example 4: Threshold instability near dosing cut-offs

Clinical question: Is pregabalin 150 mg three times daily appropriate?

The SmPC recommends dose adjustment based on creatinine clearance (Cockcroft–Gault) using defined dosing categories: ≥60 mL/min (up to 600 mg/day), 30–60 mL/min (up to 300 mg/day), 15–30 mL/min (up to 150 mg/day), and <15 mL/min (up to 75 mg/day) [61].

In this patient, estimated creatinine clearance ranges from 55 mL/min (adjusted body weight) to 77 mL/min (actual body weight), placing her across two dosing categories (30–60 vs. ≥60 mL/min).

Clinical decision: Dose selection should be individualized within this range [48]. A higher dose (e.g., 450 mg/day) may be justified if pain control is inadequate and treatment is well tolerated, whereas a lower dose (300 mg/day) is appropriate in the presence of adverse effects or when adopting a more conservative approach.

This example demonstrates how categorical dosing recommendations in SmPCs may be difficult to apply when renal function estimates fall near decision thresholds, reinforcing the need for range-based and context-dependent interpretation.

## Figures and Tables

**Table 1:** Indexed eGFR versus absolute eGFR in three patients with different body sizes.

Parameter	Patient A (Frail, Low BSA)	Patient B (Tall, Normal BSA)	Patient C (Obese, High BSA)
Sex	Female	Male	Female
Age (years)	68	55	63
Height (cm)	150	190	157
Weight (kg)	45	85	93
BMI (kg/m^2^)	20.0	23.5	37.7
Serum creatinine (mg/dL)	1.05	1.30	1.10
eGFR (CKD-EPI 2021, mL/min/1.73 m^2^)	58	65	56
BSA (Mosteller, m^2^)	1.37	2.12	2.01
Absolute eGFR (mL/min)	46	80	65

BSA, body surface area; BMI, body mass index; eGFR, estimated glomerular filtration rate.

## Data Availability

No new data were created or analyzed in this study. Data sharing is not applicable to this article.
